# L-DOPA Improves Ventilation but Not the Ventilatory Response to Hypercapnia in a Reserpine Model of Parkinson’s Disease

**DOI:** 10.3390/brainsci13050775

**Published:** 2023-05-08

**Authors:** Monika Jampolska, Kryspin Andrzejewski, Paweł M. Boguszewski, Katarzyna Kaczyńska

**Affiliations:** 1 Department of Respiration Physiology, Mossakowski Medical Research Institute, Polish Academy of Sciences, Pawińskiego 5 St., 02-106 Warsaw, Poland; mjampolska@imdik.pan.pl (M.J.); kandrzejewski@imdik.pan.pl (K.A.); 2 Laboratory of Animal Models, Neurobiology Centre, Nencki Institute of Experimental Biology of Polish Academy of Sciences, Ludwika Pasteura 3 St., 02-093 Warsaw, Poland; p.boguszewski@nencki.edu.pl

**Keywords:** Parkinson’s disease, reserpine, L-DOPA, rat model, breathing, hypercapnia

## Abstract

Parkinson’s disease (PD) is a neurological disorder characterized by progressive degeneration of the substantia nigra that affects mainly movement control. However, pathological changes associated with the development of PD may also alter respiration and can lead to chronic episodes of hypoxia and hypercapnia. The mechanism behind impaired ventilation in PD is unclear. Therefore, in this study, we explore the hypercapnic ventilatory response in a reproducible reserpine-induced (RES) model of PD and parkinsonism. We also investigated how dopamine supplementation with L-DOPA, a classic drug used to treat PD, would affect the breathing and respiratory response to hypercapnia. Reserpine treatment resulted in decreased normocapnic ventilation and behavioral changes manifested as low physical activity and exploratory behavior. The respiratory rate and the minute ventilation response to hypercapnia were significantly higher in sham rats compared to the RES group, while the tidal volume response was lower. All of this appears to be due to reduced baseline ventilation values produced by reserpine. L-DOPA reversed reduced ventilation, indicating a stimulatory effect of DA on breathing, and showed the potency of DA supplementation in restoring normal respiratory activity.

## 1. Introduction

Parkinson’s disease (PD) is a neurological disorder that affects movement control. In PD, neurons of the substantia nigra progressively degenerate; as a result, the amount of dopamine (DA) available for neurotransmission in the corpus striatum is reduced. The biochemical imbalance manifests itself with typical clinical symptoms, including resting tremor, rigidity, bradykinesia or gradual slowing of spontaneous movements, loss of postural reflexes, poor balance, and motor coordination [1,2]. Pathological conditions associated with the development of PD can affect respiratory performance. Disorders of the upper respiratory tract or muscles involved in breathing can be considered peripheral, while they can be classified as central if functional deficits involve important neurons located in the brainstem participating in breathing control [3,4]. Impaired ventilation can lead to chronic episodes of hypoxia and hypercapnia, which in turn worsens quality of life and increases overall morbidity, including deterioration of brain function. The sparse literature describing the respiratory response to hypercapnia in patients with PD is inconsistent, as a broad spectrum of changes have been observed from decline, without change to increase [5,6,7]. This diversity of observed responses to hypercapnia is difficult to interpret but may have been related to the heterogeneity of the study groups, including the stage of disease in the patients studied. Nonetheless, these findings indicate impaired chemical control of respiration, which may be related to abnormalities in the brainstem observed in idiopathic PD, such as the presence of intra-cytoplasmic α-synuclein aggregates or degenerative changes in the chemosensitive locus coeruleus [4]. So far, two studies in 6-hydroxydopamine (6-OHDA) induced animal models have shown an inconclusive effect of the hypercapnic stimulus; a slightly increased [8] and a decreased [9] response.

The mechanism that may underlie ventilation impairment in PD is unclear. Therefore, in this study, we examine the hypercapnic ventilatory response in a reproducible model induced by reserpine (RES) of PD and parkinsonism, commonly used as a predictor of the symptomatic potency of new agents [10]. Reserpine produces a wide range of motor impairments (akinesia, hypokinesia, limb rigidity, and oral tremor) as well as affective disorders (memory deficits, depressive, anxiety, and anhedonic-like behaviors) that resemble PD characteristics [11,12,13,14]. Reserpine, a specific inhibitor of the vesicular monoamine transporter (VMAT2) [15], induces loss of storage capacity and extensive depletion of not only brain DA but also serotonin (5-HT) and noradrenaline (NA) [16,17]. The role of dopamine in regulating the response to a hypercapnic stimulus has been relatively less studied and has not yet been clarified. The effect of L-DOPA medication on respiratory function is rarely studied, and to date, the effect of L-DOPA treatment on the respiratory response to hypercapnia has not been studied [4]. In this regard also, as experimental research on the respiratory response to hypercapnia in PD models is inconsistent [8,9], the purpose of this study is to examine the respiratory response to two stages of hypercapnia in a reserpine-induced PD model and investigate how this response is affected by dopamine supplementation with its precursor L-DOPA.

## 2. Materials and Methods

### 2.1. Animals

The study was approved by the local Ethics Committee for Animal Research (WAW2/134/2018). A total of nineteen conscious male adult Wistar rats were used at 10–12 weeks of age. Animals were individually housed in conventional plastic-steel cages in a 12 h light/dark cycle at a room temperature of 21 C, with commercial rodent food and water ad libitum.

### 2.2. Model and Drugs

The reserpine model was induced in rats by intraperitoneal (ip) pretreatment with reserpine and 16 h later with α-methyl-tyrosine (α-MT). Reserpine (2.5 mg/kg; Sigma Aldrich, Poznań, Poland) was dissolved in a solution of a mixture consisting of 0.25% citric acid, 2% benzyl alcohol, and 10% Tween-80. α-Methyl-tyrosine (250 mg/kg; Sigma Aldrich, Poland) was dissolved in a saline solution. Reserpine is a specific inhibitor of the vesicular monoamine transporter (VMAT2) [15], which produces a loss of storage capacity and extensive depletion of brain DA, 5-HT, and NA [16,17]. α-MT is an inhibitor of tyrosine hydroxylase, which inhibits the synthesis of DA and NA and prolongs neurochemical deficits [18]. To study the effect of DA supplementation on the hypercapnic respiratory response, L-DOPA (25 and 100 mg/kg, Sigma Aldrich, Poland), a precursor of the neurotransmitter dopamine, was dissolved in a saline solution with benserazide (6.25 mg/kg, Sigma Aldrich, Poland), an inhibitor of peripherally acting decarboxylase. All solutions were freshly prepared before each injection.

### 2.3. Ventilation Measurements

Ventilation and its responses to 4% and 7% CO_2_ in O_2_ (two hypercapnia stimuli) were studied in a rodent whole-body plethysmograph (model PLY3223; Buxco Electronix Inc., Wilmington, NC, USA). The pressure difference between the experimental and reference chambers was measured using a differential pressure transducer. The pressure signal was amplified, filtered, recorded, and analyzed with data analysis software (Biosystem XA for Windows, SFT3410 230 v2.9; Buxco Electronics, Wilmington, NC, USA) generating tidal volume (V_T_, mL) and breathing frequency (F, breaths/min). Minute ventilation (V_E_, mL min^−1^, BTPS) was determined as a product of tidal volume and breathing frequency. V_T_ and V_E_ were normalized to body weight (mL/kg and mL/kg/min, respectively). All experiments were carried out at room temperature (22–24 °C). The chamber (4.7 L) was ventilated continuously with atmospheric air at a rate of 2.5 L/min to avoid CO_2_ accumulation. Volume calibration was completed before each experiment by injecting a known volume of air into the chamber. Each rat remained unrestrained in the recording chamber for 30 min for adaptation before measurements of baseline normoxic parameters. Acute hypercapnia was achieved by rapid flushing with hypercapnic O_2_-balanced air (5 L). The rectal temperature was taken before and at the end of each experiment.

### 2.4. Open-Field Test

Open-field studies were conducted to confirm the efficacy of the reserpine model expressed in terms of motor impairment. Secondly, the effect of the dopamine precursor on motor dysfunction was studied. The test animal was placed for 15 min in an observation field (dimensions: 75 × 75 × 35 cm) illuminated with soft and diffused light. The entire session was recorded to digital video files and then automatically analyzed using image recognition software (EthoVision XT 10 and BehaActive) to count parameters describing movement (distance, moving, mobility). The three minutes of highest activity were taken for behavioral analysis.

### 2.5. Protocol

The experimental protocol that included breathing and behavioral tests involved two groups of animals:

SHAM (n = 5); reserpine solvent and α-MT were administered ip 16 h apart. Forty minutes after the injection of vehiculum and α-MT, an open-field test lasting 15 min was performed. The ventilatory response to two degrees of hypercapnia (4% and 7% CO_2_ in O_2_) was then evaluated. Subsequently, L-DOPA + benserazide was administered ip and 30 min later hypercapnic ventilatory responses and the open-field test were reexamined.

RES group (n = 14); the same sequence of actions was repeated in rats treated with reserpine + α-MT. Rats were ip injected with reserpine at a dose of 2.5 mg/kg, 16 h later α-methyl-tyrosine was given at a dose of 250 mg/kg (ip). The development of PD symptoms was checked 1 h after α-MT using an open-field test. The response to two degrees of hypercapnia (4% and 7% CO_2_ in O_2_) was then tested. The reserpine group consisted of two equal groups; each received one dose of L-DOPA, 25 mg/kg (n = 7) or 100 mg/kg (n = 7). The ip benserazide and L-DOPA were then administered and 30 min afterward hypercapnic ventilatory responses and the open-field test were reinvestigated.

### 2.6. Statistical Analysis

Statistical comparisons began with the descriptive tests of normality performed with the Shapiro–Wilk test. Data were analyzed using non-parametric statistics. The differences between the sham and RES groups were evaluated using the Mann–Whitney U test. For comparison within the group, Wilcoxon’s signed ranks test was used. All experimental data are presented as mean ± SEM. In all cases, *p* < 0.05 was considered statistically significant. Statistical analysis was performed using STATISTICA (StatSoft, Kraków, Poland).

## 3. Results

### 3.1. Behavioral Analysis

Physical activity and exploratory behavior were evaluated in control (SHAM) and reserpine-treated rats (RES). The results shown in Table 1 indicate a decrease in locomotor activity in the animals after reserpine administration. The distance covered by a healthy rat is characterized by exploration of the entire area of the open field, both the center and the perimeter of the arena. RES rats showed no movement in the center of the open field, without leaving the center of the arena throughout the test. RES rats, compared to SHAM, covered virtually no distance, which may indicate the animals’ inability to move due to muscle stiffness. At the same time, it confirms the effectiveness of reserpine in modeling PD. Only the higher dose of L-DOPA 100 mg/kg induced significant changes in distance, mobility, and movement compared to reserpine rats without L-DOPA and treated with a lower dose of the compound (Table 1).

### 3.2. Normocapnic Breathing and Respiratory Response to Hypercapnia after Reserpine Treatment

**Figure 1:**
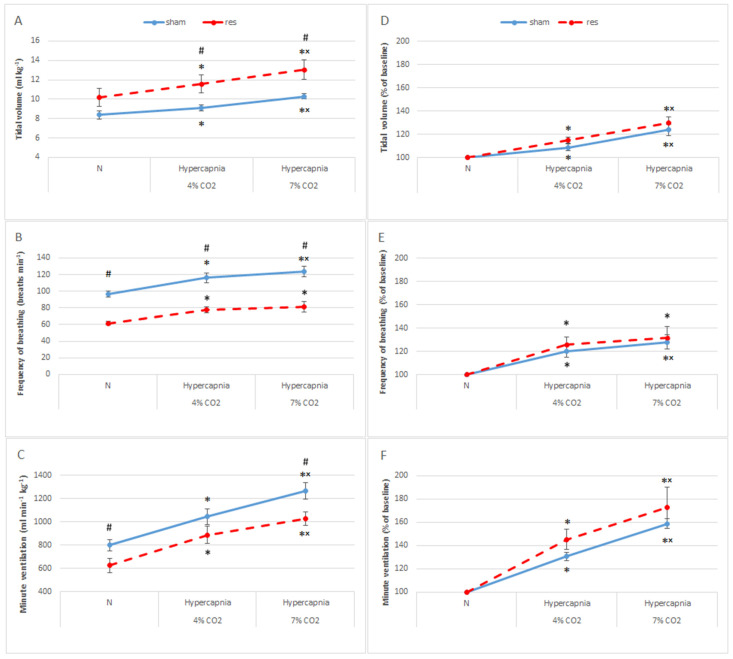
Tidal volume (**A**), frequency of breathing (**B**), and minute ventilation (**C**) during air breathing (normocapnia) and ventilatory response to hypercapnia in sham (blue line) and reserpine-treated rats (red line). Tidal volume (**D**), frequency of breathing (**E**), and minute ventilation (**F**) reactivity to hypercapnia expressed as a percentage of baseline (normocapnia). The data are presented as mean ± SEM; * *p* < 0.05 vs. normocapnia value, ^×^ *p* < 0.05 vs. 4% CO_2_ response, # *p* < 0.05, vs. sham or reserpine group.

During normocapnic respiration, the tidal volume of the rats in the reserpine group increased compared with the control parameters of sham rats (Figure 1A). On the contrary, there was a significant 40% reduction in respiratory rate, which resulted in a 25% decrease in minute ventilation in the RES group (Figure 1B,C). The respiratory response to two-stage hypercapnia retained its classic character in both groups of rats, with the stronger stimulus producing a stronger hyperventilation response (Figure 1). The respiratory rate and the minute ventilation response to hypercapnia were significantly higher in sham rats compared to the RES group, while the tidal volume response was lower. All of this appears to be due to altered baseline ventilation values produced by reserpine.

To see the responsiveness of all respiratory parameters to hypercapnia in both groups of test animals, data were expressed as a percentage of the baseline. All ventilation parameters in the RES group reached a higher level of reactivity to the hypercapnia stimulus, but the difference did not reach statistical significance compared to the sham group (Figure 1D–F). Despite reduced normocapnic minute ventilation in RES rats, 4% CO_2_ increased minute ventilation by 45% and 7% CO_2_ by 72% (Figure 1D–F). Sham rats showed reduced responsiveness to hypercapnia and responded with a smaller increase in minute ventilation of 31% and 59%, respectively.

### 3.3. Normocapnic Breathing and Respiratory Response to Hypercapnia in RES Rats after L-DOPA Treatment

**Figure 2:**
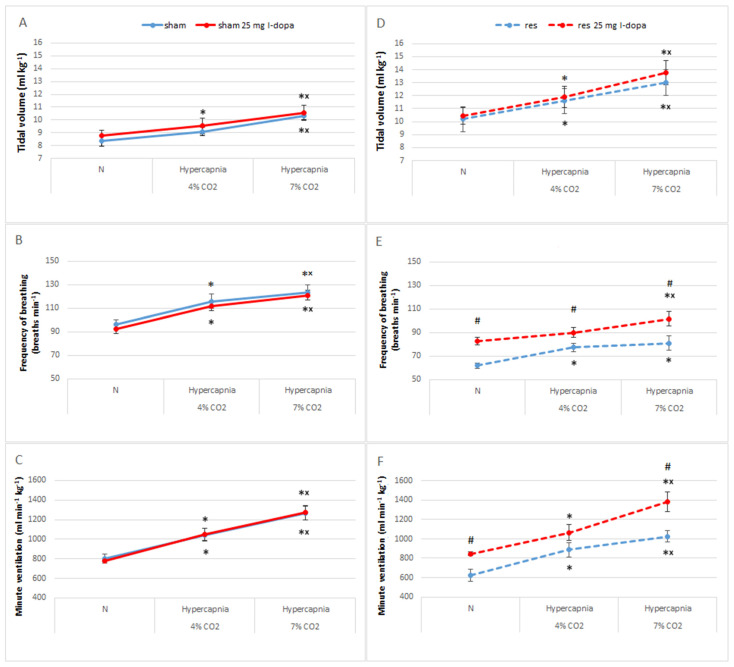
Tidal volume (**A**), frequency of breathing (**B**), and minute ventilation (**C**) during air breathing (normocapnia) and ventilatory response to hypercapnia in sham rats (blue line) treated with L-DOPA (red line). Tidal volume (**D**), frequency of breathing (**E**), and minute ventilation (**F**) during air breathing (normocapnia) and ventilatory response to hypercapnia in reserpine rats (blue dashed line) treated with L-DOPA (red dashed line). The data are presented as mean ± SEM; * *p* < 0.05 vs. normocapnia value, ^×^ *p* < 0.05 vs. 4% CO_2_ response, ^#^ *p* < 0.05, vs. reserpine group.

Treatment with L-DOPA + benserazide in the sham group had no effect on either baseline respiratory parameters or their stimulation during the hypercapnic respiratory response (Figure 2A–C).

In contrast, in the reserpine group, L-DOPA (25 mg/kg) significantly increased normocapnic respiratory rate values and, consequently, their response to two-stage hypercapnia. This also translated into an increase in minute ventilation during normocapnia and hypercapnia, despite no change in the values of the tidal volume (Figure 2D–F). It appears that the increased level of hypercapnic ventilatory response after L-DOPA in RES rats is simply due to the initially elevated baseline normocapnic ventilation by the DA precursor.

**Figure 3:**
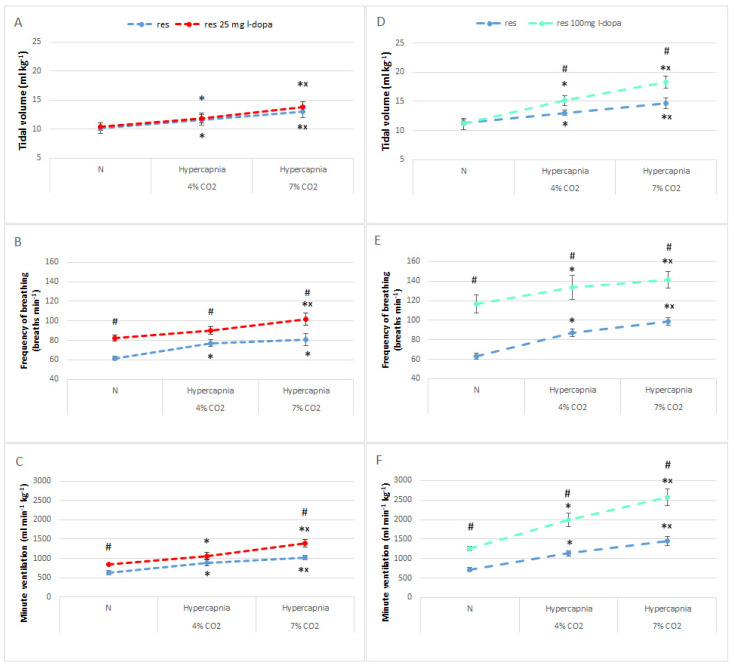
Tidal volume (**A**,**D**), frequency of breathing (**B**,**E**), and minute ventilation (**C**,**F**) during air breathing (normocapnia) and ventilatory response to hypercapnia in reserpine rats (blue dashed line) treated with L-DOPA; 25 mg/kg (red dashed line) and 100 mg/kg (green dashed line). The data are presented as mean ± SEM; * *p* < 0.05 vs. normocapnia value, ^×^ *p* < 0.05 vs. 4% CO_2_ response within the same group, ^#^ *p* < 0.05, vs. reserpine groups.

In further steps, we decided to test the effect of a higher dose of L-DOPA (100 mg/kg) on the animals’ basal ventilation and ventilatory response to hypercapnia. The lower dose of 25 mg/kg stimulated only normocapnic respiration, which is the frequency of breathing and minute ventilation (Figure 3A–C), and had no effect on animal behavior. Therefore, we hypothesized that a higher dose, affecting behavioral changes in rats may more significantly impact respiration including the respiratory response to hypercapnia. In fact, 100 mg of L-DOPA significantly increased, almost doubling, the normocapnic values of basic respiratory parameters such as respiratory rate and minute ventilation in reserpine rats (Figure 3D–F). The baseline normocapnic tidal volume remained unchanged regardless of the dose of L-DOPA used (Figure 3A,D).

**Figure 4:**
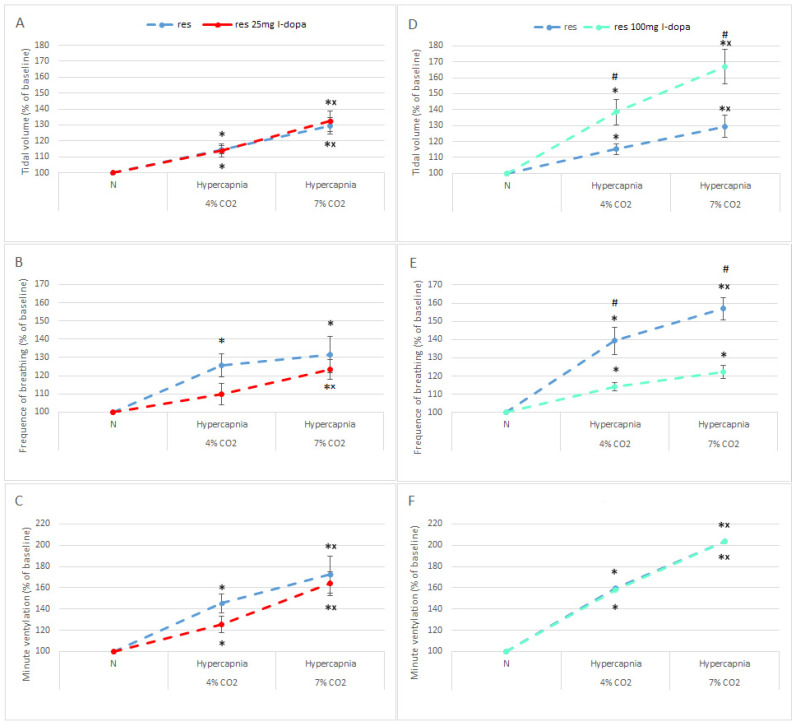
Tidal volume (**A**,**D**), frequency of breathing (**B**,**E**), and minute ventilation (**C**,**F**) reactivity to hypercapnia expressed as a percentage of baseline (normocapnia) in reserpine rats (blue dashed line) treated with L-DOPA; 25 mg/kg (red dashed line) and 100 mg/kg (green dashed line). The data are presented as mean ± SEM; * *p* < 0.05 vs. normocapnia value, ^×^ *p* < 0.05 vs. 4% CO_2_ response within the same group, ^#^ *p* < 0.05, vs. response without L-DOPA treatment.

To test whether L-DOPA injection affects respiratory reactivity to hypercapnia, rather than just baseline values, data were expressed as a percentage of initial normocapnic values. As shown in Figure 4, a smaller dose of L-DOPA did not increase respiratory reactivity to the hypercapnic stimulus, which is even lower than before treatment in the case of respiratory rate and minute ventilation response, but insignificantly (Figure 4A–C). In the case of 100 mg of L-DOPA, the effect was more profound compared to the 25 mg dose. Above all, tidal volume reactivity to hypercapnia increased significantly above 60% only after 100 mg of L-DOPA (Figure 4A,D).

Although 100 mg of L-DOPA significantly increased the basal respiratory rate (Figure 3E), its reactivity in response to hypercapnia was significantly reduced by approximately 30% compared to the control reserpine group (Figure 4E). This is probably due to the significantly doubled basal respiratory rate after the DA precursor, which reaches a certain threshold and cannot increase further in response to hypercapnia. The increased tidal volume reactivity compensated for the reduced respiratory rate reactivity, producing an unchanged minute ventilation reactivity (Figure 4F). That is, the main effect of L-DOPA on respiration is dose-dependent and is related to stimulation of normocapnic respiration without affecting hypercapnic responsiveness.

## 4. Discussion

The main findings of this study showed that rats treated with reserpine exhibit reduced lung ventilation in normocapnia and a parallel reduced ventilatory response to hypercapnia. L-DOPA treatment restored ventilation in PD rats to the level observed in the control group with a dose of 25 mg and significantly magnified it above the control level with 100 mg, but neither dose increased further hypercapnia responsiveness. This indicates that DA, present in almost all regions of the bulbar respiratory network [19,20,21,22], plays an important role in mediating normocapnic air-breathing respiration. Dopamine and dopaminergic neurons have previously been shown to be involved in mediating respiration in normoxia and hypoxia. Dopaminergic neurons or dopamine depletion in a PD model or in humans results in an impaired breathing pattern [23,24] and altered, usually diminished, reactivity to hypoxia [23,25,26,27].

The function of dopamine, on the other hand, in regulating the response to a hypercapnic stimulus has been relatively less studied and has not yet been elucidated. Data on the effect of dopamine on the response to hypercapnia are inconsistent. One study reported a significant increase in the respiratory response to CO_2_ under the influence of apomorphine (APO), a non-selective DA agonist [28], while another found a decrease in the respiratory rate response and an increase in the tidal volume response [29]. Subsequent work showed a significantly lowered hypercapnic respiratory rate but without a significant change in minute ventilation in DAT knockout mice, characterized by functional hyperdopaminergia [30]. In our study, the hypercapnic respiratory response in RES rats, expressed as a percentage of their baseline value, indicates a trend toward increased responsiveness compared to the sham group, but the increase is insignificant. On the other hand, administration of a high dose of L-DOPA resembles the hyperdopaminergic state observed in the study by Vincent et al., 2007 [30], and very similar effects, i.e., an increase in tidal volume reactivity, a decrease in frequency, and consequently no change in minute ventilation in response to increased CO_2_.

Sparse reports that examined the response to a hypercapnic stimulus in a 6-OHDA- induced rat model of PD, characterized by depletion of dopamine neurotransmission, documented a slight increase in ventilation, explained by unilateral lesion, and compensation coming from the intact hemisphere [8], and in another study, a reduction associated with loss of neurons in the ventral respiratory column of the medulla [9]. However, note that in our model, reserpine causes more than just DA depletion. Reserpine is an alkaloid that irreversibly blocks the neuronal vesicular monoamine transporter that inhibits the uptake and storage of monoamine neurotransmitters such as DA, NA, and 5-HT in the synaptic vesicles of neurons [31]. As we showed in our earlier publication in this model, in addition to a 75% loss of DA, substantial deficits in 5-HT (72%) and NA (92%) are observed in the brainstem [17] and the response to hypercapnia may be the resultant loss of all three amines.

As shown in previous studies, damage to noradrenergic neurons performed by injection of 6-OHDA into the brainstem chemosensitive locus coeruleus [32] or depletion of bulbospinal catecholaminergic cells by injection of the immunotoxin anti-dopamine β-hydroxylase-saporin via thoracic spinal cord injection (lesioned C1 and A5) or through the fourth ventricle (lesioned A5, A6, A7, C1, and C2), results in a reduced respiratory response to hypercapnia [33,34]. Destruction of serotonergic neurons of the chemosensitive raphe nuclei with an injection of a conjugate of a monoclonal antibody into the serotonin transporter (SERT) and saporin also decreases the respiratory response to increased CO_2_ in inhaled air [35]. On the contrary, a much smaller loss of serotonin of approximately 30% in the striatum and 20% in the brainstem induced by an injection of 6-OHDA into MFB resulted in an increased response to hypoxia. Unfortunately, the response to hypercapnia was not studied [36]. A recent paper indicated a crucial role for 5-HT in aged mice with brain serotonin shortages, associated with tryptophan hydroxylase 2 deficiency (*Tph2*), which had reduced sensitivity to hypercapnia [37]. However, the young mice were able to maintain an unchanged respiratory response to hypercapnia compared to their controls while awake.

Central activation of dopamine receptors has a stimulating effect on the respiratory response to CO_2_, as described previously [27,28,38]. Thus, the loss of DA in the brainstem should have an inhibitory effect on the response to hypercapnia. However, the response to hypercapnia in reserpine rats appears to remain unchanged, which may indicate some tendency to compensate for the loss of all three biogenic amines. It is remarkable that with such a significant loss of all three amines, reserpine rats were able to maintain a similar level of response to hypercapnia as sham rats despite significantly reduced basal minute ventilation.

To replenish DA deficiency in the brain of RES rats, we administered L-DOPA with benserazide to block its peripheral conversion to DA and intensify the central production of DA from L-DOPA. In fact, L-DOPA was able to magnify the reduced normocapnic ventilation present in the reserpine group, but the reactivity to hypercapnia was not altered. At the same time, L-DOPA did not show an effect on breathing in the sham group, which was in agreement with a previous study [38]. We tested a dose of 25 mg/kg on the suggestion of previous studies, including the effect of levodopa on the respiratory response to hypoxia [39,40], and this dose proved sufficient to reverse the respiratory depression present in our reserpine rats but without further stimulation of the hypercapnic response. Even the four-fold higher dose used in our research, while significantly raising normocapnic ventilation, had no effect on hypercapnia responsiveness, as shown in a study in healthy mice [38]. We can also speculate that with the simultaneous loss of three important respiratory mediators, such as DA, 5-HT, and NA, DA supplementation alone may not be powerful enough to stimulate CO_2_ reactivity in our study.

Our behavioral test confirmed that reserpine treatment in rats models the main features of PD, such as akinesia, bradykinesia, and rigidity, as manifested by their dramatic immobility. The smaller dose of L-DOPA that we used was not effective in reversing the motor symptoms of PD, corresponding to a previous study in which doses of 25 and 50 mg/kg were ineffective only until a dose of 100 mg/kg significantly increased locomotor activity in rats treated with reserpine [40]. The latter was also confirmed by our study, where 100 mg/kg of L-DOPA simultaneously improved motor changes and profoundly stimulated respiration. Interestingly, if the 25 mg/kg dose of L-DOPA used in our study restores normal ventilation, but without affecting behavioral activity, this means that the decrease in ventilation was not a consequence of chest muscle stiffness, but was rather associated with a central disruption of DA neurotransmission.

However, the study has some limitations that point to future research directions. It would be worthwhile to investigate, in this model, the role and effects of other amines, i.e., 5-HT and NA, using their precursors and activators, in the regulation of respiration, including the response to hypercapnia.

## 5. Conclusions

In conclusion, the data presented here show that reserpine treatment, which produces depletion of DA, 5-HT, and NA and symptoms of parkinsonism, results in depressed lung ventilation in normocapnia and hypercapnia conditions. The lower response to hypercapnia is due to lower levels of control breathing since the reactivity of this response is at the same level as in sham animals. L-DOPA reverses reduced ventilation, indicating a stimulating effect of DA on normocapnia breathing and showing the potency of DA supplementation in restoring normal respiratory activity.

## Figures and Tables

**Table 1:** Parameters of mobility and exploration monitored during the open-field test.

	Distance (cm)	Moving (s)	Mobile (s)
SHAM	554 ± 73	62 ± 8.8	58 ± 6.8
SHAM + L-DOPA	420 ± 93	47 ± 9.2	48 ± 8.3
RES	1.62 ± 0.33 ^##^	0.08 ± 0 ^#^	0.08 ± 0 ^#^
RES + L-DOPA 25 mg	1.58 ± 0.73 ^##^	0.18 ± 0.06 * ^#^	0.25 ± 0.17 ^##^
RES + L-DOPA 100 mg	32 ± 8.2 ** ^## ++^	2.28 ± 1.0 * ^#^	8.2 ± 3.25 ** ^## +^

Statistical differences; * *p* < 0.05, ** *p* < 0.001 vs. corresponding value of RES group, ^#^ *p* < 0.05, ^##^ *p* < 0.001 vs. corresponding value of SHAM groups, and ^+^ *p* < 0.05, ^++^ *p* < 0.001 vs. RES + L-DOPA 25 mg.

## Data Availability

The data used to support the findings of this study are available from the corresponding author upon request.
